# Altered Retinal Dopamine Homeostasis in Murine Models of Retinitis Pigmentosa

**DOI:** 10.1111/jnc.70552

**Published:** 2026-09-18

**Authors:** Katri Vainionpää, Eerik Lappalainen, Anna Nilsson, Ida Pavela, Tommi Torsti, Umair Seemab, Elina Mäkinen, Reza Shariatgorji, Per E. Andrén, Aaro Jalkanen, Markus M. Forsberg, Henri Leinonen

**Affiliations:** ^1^ School of Pharmacy, Faculty of Health Sciences University of Eastern Finland Kuopio Finland; ^2^ Department of Pharmaceutical Biosciences, Spatial Mass Spectrometry, Science for Life Laboratory Uppsala University Uppsala Sweden; ^3^ Wits Integrated Molecular Physiology Research Initiative, Department of Physiology, Faculty of Health Sciences, School of Biomedical Sciences University of the Witwatersrand Johannesburg South Africa

## Abstract

Retinitis pigmentosa (RP) is a heterogeneous group of currently untreatable inherited retinal degenerations that share similar disease manifestations despite distinctive genetic causes. Dopamine (DA) is an important and tightly regulated catecholaminergic neurotransmitter required for multiple modulatory functions throughout the central nervous system, including the retina. Dysregulation of DA homeostasis has been reported during neurodegeneration, but how it is regulated in RP remains poorly understood. Here, we demonstrate that early RP is associated with marked dysregulation of the dopaminergic system using two distinct disease models, P23H and rd10 mice. We found increased DA levels in RP retinas collected from pre‐weaned (P12), juvenile (P30), and adult (P60–90) mice by utilising ultra‐high‐performance liquid chromatography and matrix‐assisted laser desorption/ionisation mass spectrometry. Consistently, DA levels were also elevated in the vitreous of P23H mice, where it likely diffuses from the retina. RP retinas additionally demonstrated higher levels of DA precursor l‐3,4‐dihydroxyphenylalanine (L‐DOPA), as well as upregulated tyrosine hydroxylase gene expression, which suggests elevated DA synthesis. In parallel, we observed increased activity and expression of the catecholamine‐metabolising enzyme catechol‐O‐methyltransferase (COMT) during retinal degeneration. Comparative tissue analysis with cortex, striatum, retina, and eye cup samples further highlighted COMT as a major contributor to catecholamine inactivation in the mouse retina. Finally, RNA sequencing of P23H retinal extracts revealed widespread alterations related to the catecholaminergic system during disease progression, including upregulation of the solute carrier family 6 member 2 gene, encoding norepinephrine transporter, and the protein phosphatase 1 regulatory subunit 1B gene, which encodes DA‐ and cAMP‐regulated neuronal phosphoprotein. In conclusion, these data demonstrate elevated DA levels in P23H and rd10 mouse retinas, along with several other biochemical changes in the catecholamine system, suggesting a hyperexcited state in early RP progression.

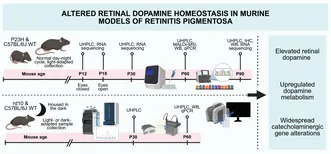

## Graphical abstract

Dopamine is an important catecholamine chemical messenger in the light‐sensing retina of the eye, where it serves multiple modulatory functions related to vision. This research studied the changes in the retinal dopamine system in two mouse models (P23H and rd10) of retinitis pigmentosa, which is a disease group of inherited retinal diseases that lead to progressive loss of vision. Measurements of the retina revealed elevated dopamine levels and metabolism in the disease model mice compared to tissue samples from healthy mice. Furthermore, analysis of gene expression revealed multiple changes in genes related to the dopaminergic and wider catecholaminergic system. Altogether, the results show that retinal catecholamine and, in particular, dopamine homeostasis is disrupted during retinal degeneration. This suggests that changes in dopamine may play an important role in the progression of disease in retinitis pigmentosa.

**Abbreviations**
6‐OHDA: 6‐hydroxydopamine
AADC: aromatic L‐amino acid decarboxylase
ADRA1/ADRA2: adrenergic receptor alpha 1/alpha 2
AM: ante meridiem
ANOVA: analysis of variance
BCA: bicinchoninic acid (protein assay)
cAMP: cyclic adenosine monophosphate
cDNA: complementary DNA
cGMP: cyclic guanosine monophosphate
COMT: catechol‐O‐methyltransferase
CTX: cortex
DA: dopamine
DAC: dopaminergic amacrine cell
DAPI: 4′,6‐diamidino‐2‐phenylindole
DARPP‐32: dopamine‐ and cAMP‐regulated neuronal phosphoprotein
DAT: dopamine transporter
DEG: differentially expressed gene
DHBAc: 3,4‐dihydroxybenzylacetic acid
DOPAC: 3,4‐dihydroxyphenylacetic acid
DRD1/DRD2/DRD4: dopamine receptor D1/D2/D4
EDTA: ethylenediaminetetraacetic acid
EtOH: ethanol
FC: fold change
FDR: false discovery rate
GEO: Gene Expression Omnibus
GPCR: G protein‐coupled receptor
H&E: haematoxylin and eosin
HPMC: hydroxypropyl methylcellulose
HRP: horseradish peroxidase
HVA: homovanillic acid
MALDI‐MSI: matrix‐assisted laser desorption/ionisation mass spectrometry imaging
MAO‐A/MAO‐B: monoamine oxidase A/B
MB‐COMT: membrane‐bound catechol‐O‐methyltransferase
MS: mouse
NDS: normal donkey serum
NET: norepinephrine/noradrenaline transporter
PBS/PBST: phosphate‐buffered saline/PBS with Tween 20
PCR: polymerase chain reaction
PFA: paraformaldehyde
PKA: protein kinase A
PVP: polyvinylpyrrolidone
qPCR: quantitative polymerase chain reaction
RB: rabbit
ReLayS: retinal layer separation
RET: retina
RNA‐seq: RNA sequencing
RP: retinitis pigmentosa
RPE: retinal pigment epithelium
RRID: Research Resource Identifier
S‐COMT: soluble catechol‐O‐methyltransferase
SD: standard deviation
STR: striatum
TBST/TBS: tris‐buffered saline with Tween/Tris‐buffered saline
TH: tyrosine hydroxylase
UHPLC: ultra‐high‐performance liquid chromatography
VA: vanillic acid
WB: western blotting
WT: wild‐type

## 1 Introduction

Retinitis pigmentosa (RP) is a group of currently untreatable inherited retinal degenerations that affect approximately 1 in 4000 people worldwide (Hartong et al. 2006). RP leads to progressive loss of eyesight beginning with night‐vision deficits due to distinct genetic mutations that primarily compromise rod photoreceptor cell function and survival. As the disease progresses, cone photoreceptor cells responsible for colour and high acuity vision also degenerate. The severity and rate of progression depend on the causal mutation at a population level, and inter‐subject variability tends to be significant. However, different mutations share similar pathological mechanisms downstream of the affected protein, including inflammation, oxidative and endoplasmic reticulum stress, and apoptosis (Newton and Megaw 2020) that provide opportunities for gene‐agnostic therapeutic approaches.

Retinal dopamine (DA) has multiple, mainly modulatory roles in all cell types of the tissue via both D1‐ and D2‐like receptors (reviewed in Djamgoz et al. 1997; Popova 2020; Witkovsky 2004). The major roles of DA include eye development, light adaptation, gap‐junctional coupling and circadian rhythms of the retina. The synthesis of DA from tyrosine and L‐3,4‐dihydroxyphenylalanine (L‐DOPA) via tyrosine hydroxylase (TH) and aromatic‐L‐amino‐acid decarboxylase (AADC), respectively, occurs in specific dopaminergic amacrine cells (DACs) located in the inner retina, from where it can diffuse throughout the retinal thickness. Both rods and cones as well as melanopsin‐containing retinal ganglion cells influence DACs and the release of DA (Cameron et al. 2009; Pérez‐Fernández et al. 2019; Qiao et al. 2016; Zhao et al. 2017), although rods have been suggested as the primary modulator of DA release (Pérez‐Fernández et al. 2019). In the retina, DA release by exocytosis is induced in the light, and presynaptic DRD2 autoreceptors participate in regulating both synthesis and release (Popova 2020; Sibley 1999). DA turnover is regulated by inactivation via multiple metabolic pathways involving monoamine oxidases A and B (MAO‐A, MAO‐B), catechol‐O‐methyltransferase (COMT), and aldehyde dehydrogenase enzymes, which generate the main metabolites 3,4‐dihydroxyphenylacetic acid (DOPAC) and homovanillic acid (HVA) as well as other intermediate metabolites (Meiser et al. 2013).

Since DA signalling is important for normal retinal function, disruptions in this system may play a role in retinal degeneration, as suggested by findings that pharmacological modulation of the dopaminergic system can influence the disease in multiple experimental settings. Specifically, pharmacological activation of D2‐like receptors with pramipexole, ropinirole, and bromocriptine has shown protective efficacy in retinal degeneration and RP models in vivo (Leinonen et al. 2019, 2024; Orban et al. 2018; Shibagaki et al. 2015; Tanaka et al. 2021). On the other hand, chemical DA depletion using 6‐hydroxydopamine (6‐OHDA), as well as both DRD1 and DRD2 antagonism, has protected rapidly degenerating rd1 retinas in organ cultures (Ogilvie and Speck 2002). The mechanisms underlying these partially paradoxical therapeutic effects are unclear. Although alterations of the dopaminergic system have been reported in dystrophic retinas (Atkinson et al. 2013; Leinonen et al. 2024; Nir et al. 2000, 2001; Nir and Iuvone 1994; Park et al. 2013; Vugler et al. 2007), systematic description of DA homeostasis during RP progression is still lacking. Therefore, this study aimed to characterise the retinal dopaminergic system during early phases of RP progression using the translationally relevant P23H and rd10 mouse models.

## 2 Materials and Methods

### 2.1 Animals and Sample Collection

Heterozygous P23H rhodopsin‐mutated mice (hereafter P23H mice, The Jackson Laboratory, strain #017628, RRID:IMSR_JAX:017628), homozygous rd10 mice carrying the phosphodiesterase 6B *Pde6b* ^R560C/R560C^ mutation (The Jackson Laboratory, strain #004297, RRID:IMSR_JAX:004297; our mouse colony originates from Institut de la Vision, Paris, France) and age‐matched wild‐type (WT) C57BL/6J (The Jackson Laboratory, strain #000664, RRID:IMSR_JAX:000664) mice were used in this study. P23H and WT mice were bred and housed in the Laboratory Animal Center, Kuopio, Finland, in a controlled environment (temperature 21°C ± 1°C, humidity 50%–60%, light period 07:00–19:00). rd10 mice were bred and housed in similar conditions but in a dark room and handled under dim red light during husbandry duties. Food and water were available *ad libitum*. Experiments with P23H mice were carried out at the ages of postnatal day (P)12, when the eyes were still unopened, and after eye‐opening at P18, and 1 (~P30), 2 (~P60) and 3 (~P90) months, whereas experiments with rd10 mice were conducted with 1‐ and 2‐month‐old mice (Table 1).

**TABLE 1:** Sample size (n) table for each main figure panel, stratified by age and experimental group.

Figure	WT	P23H	WT	rd10
1A UHPLC (batch)	P12 *n* = 8; P18 *n* = 7; P30 *n* = 4; P60 *n* = 10	P12 *n* = 8; P18 *n* = 9; P30 *n* = 7; P60 *n* = 12		
1B UHPLC (pooled)	P12 *n* = 8; P18 *n* = 7; P30 *n* = 10, P60‐90 *n* = 26	P12 *n* = 8; P18 *n* = 9; P30 *n* = 17; P60‐90 *n* = 28		
1C UHPLC	P60 *n* = 8	P60 *n* = 8		
1D UHPLC (batch)	P12 *n* = 3; P18 *n* = 7; P30 *n* = 4; P60 *n* = 4	P12 *n* = 7; P18 *n* = 9, P30 *n* = 7; P60 *n* = 6		
1E UHPLC (pooled)	P12 *n* = 8; P18 *n* = 7; P30 *n* = 10; P60 *n* = 20	P12 *n* = 8; P18 *n* = 9, P30 *n* = 16; P60 *n* = 22		
1F UHPLC (pooled)	P12 *n* = 8; P18 *n* = 7; P30 *n* = 10; P60 *n* = 16	P12 *n* = 8; P18 *n* = 9, P30 *n* = 16; P60 *n* = 16		
1G MALDI‐MSI	P60 *n* = 5	P60 *n* = 5		
2A UHPLC (pooled)			LA P30 *n* = 4; LA P60 *n* = 10; DA P60 *n* = 5	LA P30 *n* = 7; LA P60 *n* = 20; DA P60 *n* = 10
2B UHPLC (pooled)			LA P30 *n* = 4; LA P60 *n* = 11; DA P60 *n* = 4	LA P30 *n* = 7; LA P60 *n* = 18; DA P60 *n* = 9
2C UHPLC (pooled)			LA P30 *n* = 4; LA P60 *n* = 10; DA P60 *n* = 4	LA P30 *n* = 7; LA P60 *n* = 18; DA P60 *n* = 9
3A qPCR	P60 CTX *n* = 6; STR *n* = 6; RET *n* = 5; EC *n* = 6			
3B‐E qPCR & 3F‐H WB	P60 CTX *n* = 6; STR *n* = 6; RET *n* = 6; EC *n* = 6			
3I WB	P60 CTX *n* = 6; STR *n* = 6; RET *n* = 6; EC *n* = 5			
4A‐B UHPLC	P12 *n* = 7; P18 *n* = 7; P30 *n* = 4; P60 *n* = 4	P12 *n* = 8; P18 *n* = 8, P30 *n* = 6; P60 *n* = 6		
4C UHPLC			LA P30 *n* = 4; LA P60 *n* = 6	LA P30 *n* = 7; LA P60 *n* = 10
4D qPCR	P60 *n* = 4	P60 *n* = 7	LA P60 *n* = 4	LA P60 *n* = 4
4E WB	P90 *n* = 7	P90 *n* = 12	LA P60 *n* = 8	LA P60 *n* = 12
5A qPCR	P60 *n* = 4	P60 *n* = 7	LA P60 *n* = 4	LA P60 *n* = 4
5B qPCR	P60 *n* = 4	P60 *n* = 6	LA P60 *n* = 3	LA P60 *n* = 3
5E‐F WB	P90 *n* = 10	P90 *n* = 17	LA P60 *n* = 8	LA P60 *n* = 12
5G UHPLC	P90 *n* = 10	P90 *n* = 10		
5H UHPLC			LA P60 *n* = 5; DA P60 *n* = 5	LA P60 *n* = 10; DA P60 *n* = 10
6 RNA‐seq	P12 *n* = 5; P18 *n* = 5; P30 *n* = 7; P90 *n* = 3	P12 *n* = 6; P18 *n* = 6; P30 *n* = 7; P90 *n* = 4		

*Note:* Supporting Information S1 file includes source data and outlier exclusions.

Abbreviations: CTX = cortex, DA = dark‐adapted, EC = eye cup, LA = light‐adapted, MALDI‐MSI = matrix‐assisted laser desorption/ionisation mass spectrometry imaging, P = postnatal day, qPCR = quantitative polymerase chain reaction, RET = retina, RNA‐seq = RNA sequencing, STR = striatum, UHPLC = ultra‐high performance liquid chromatography, WB = western blotting, WT = wild‐type.

For most of the experiments, animals were euthanized with CO_2_, and death was confirmed by cervical dislocation. Fresh retinas were collected by enucleating the mouse eye and inserting the blunt side of micro‐dissection scissors between the retina and the sclera/retinal pigment epithelium (RPE) layer through the opening of the optic nerve. The pigmented tissue was then cut in four directions, and the surrounding eye structures around the retina were gently peeled away before the final separation of the retina from the remaining lens and vitreous. For the collection of brain tissues, mice were overdosed with ketamine‐medetomidine (Ketaminol Vet 0.1 mg/mL, MSD, Domitor Vet 7.5 mg/mL, Orion Pharma) and blood was flushed out by transcardial perfusion with ice‐cold saline for 3 min, and the tissues (cortex, striatum, retina and eye cup) were collected. Animals were typically light‐adapted during sample collection, except for a subset of rd10 mice and their WT controls, as those fresh retina samples for ultra‐high‐performance liquid chromatography (UHPLC) were collected from dark‐adapted mice under red dim light. Sample collection took place in the morning (7–10 AM). The experiments were conducted according to the Council of Europe (Directive 2010/63/EU) and Finnish guidelines and approved by the Animal Experiment Board of Finland (ESAVI‐26320‐2021, ESAVI‐2024‐0037754).

### 2.2 Ultra‐High‐Performance Liquid Chromatography

Fresh retina samples were dissected from the eye, weighed and snap‐frozen in liquid nitrogen for UHPLC analysis. Samples were homogenised in 10× ice‐cold MilliQ water (1 mg = 10 μL) by ultrasound sonication, and the homogenates were divided into two equal aliquots. The colour of the homogenate was visually assessed to evaluate if RPE contamination (black homogenate colour) had occurred during the tissue extraction. For the quantification of DA, L‐DOPA and DOPAC, 25× volume of ice‐cold 0.1 M perchloric acid (Merck, catalogue number MC1005170250) was added to one aliquot and the sample was thoroughly mixed and incubated on an ice‐bed for 10 min. A volume of 50× of ice‐cold MilliQ water was added before centrifugation at 15000*g* for 20 min at 4°C, and the supernatant was filtered (Acrodisc Syringe Filter wwPTFE 0.2 μm, 13 mm, Pall Corporation, catalogue number 513‐0165) and transferred to an UHPLC vial. For vitreous measurements, vitreous was collected through the ora serrata with a 27 G insulin needle and 3 μL of sample were homogenised in 21 μL of 0.1 M perchloric acid, then mixed with 144 μL of MilliQ before centrifugation and filtering the supernatant. UHPLC analysis was conducted in a randomised sample order with Antec Alexys Monoamine Analyser (Antec Scientific) with a 10 μL injection volume and two channels (1. catecholamines: DA; 2. metabolites: L‐DOPA, DOPAC). Specifications for UHPLC measurements are listed in Table 2. Clarity Chromatography Software (DataApex) was used for instrument control and data quantification. Analyte concentrations were calculated from integrated peak areas with the use of linear (*r* ^2^ > 0.997) calibration curves. DA (Sigma‐Aldrich, catalogue number H8502‐5G), L‐DOPA (Sigma, D‐9628) and DOPAC (Sigma‐Aldrich, 850217‐1G) standard curves ranged from 0.25 to 75 nM.

**TABLE 2:** Specifications for ultra‐high‐performance liquid chromatography experiments.

Channel	1. Catecholamines	2. Metabolites	3. COMT activity measurement
C18 Column (kept at 35°C)	NeuroSep 105, 50 mm x 1.0 mm, 3 μm, Antec Scientific	ALF‐115, 150 mm x 1.0 mm, Antec Scientific	ALF‐115, 150 mm x 1.0 mm, Antec Scientific
Cell potential	0.46 V	0.8 V	0.95 V
Flow rate	50 μL/min	75/85 μL/min	50 μL/min
Pressure	52 bar	194 bar	194 bar
Liquid phase	50 mmol/L phosphoric acid (Honeywell, cat. no. 79606‐500ML), 0.1 mmol/L EDTA (Merck, 1 084 180 250), 600 mg/L octane sulfonic acid (Sigma‐Aldrich, O0133‐100G), 6% v/v acetonitrile (VWR, 83640.320), pH 6.0	50 mmol/L phosphoric acid, 0.1 mmol/L EDTA, 600 mg/L octane sulfonic acid, 6% v/v acetonitrile, citric acid 10.51 g/L (Sigma‐Aldrich, 33114‐500G), pH 3	50 mmol/L phosphoric acid, 0.1 mmol/L EDTA, 600 mg/L octane sulfonic acid, 6% v/v acetonitrile, citric acid 10.51 g/L, pH 3

For the COMT activity assay, the second aliquot was combined with an equal volume of 20 mM sodium phosphate buffer (pH 7.4) containing 1 mM dithiothreitol (Invitrogen, catalogue number D1532). Next, the samples were mixed and centrifuged at 900*g* for 10 min at 4°C and the supernatants were collected. A pooled solution of enzyme reaction reagents was prepared in MilliQ water with 0.1 M sodium phosphate, 200 μM S‐adenosyl‐L‐methionine (Sigma‐Aldrich, catalogue number A4377), 5 mM magnesium chloride (VWR, 25107.292) and 240 μM 3,4‐dihydroxybenzylacetic acid (DHBAc) substrate (Sigma‐Aldrich, 37 580‐25G‐F). For blank samples, a similar solution was prepared but without DHBAc. Sample supernatant (10 μL) was incubated with 15 μL of either reaction or blank solution for 30 min at 37°C. The reaction was halted with 2.5 μL of 4 M perchloric acid, and the sample was diluted 1:10 with MilliQ water, centrifuged at 5400 *g* for 10 min at 4°C, filtered, and finally diluted 1:2 with MilliQ water before UHPLC. The amount of vanillic acid (VA) formed during the reaction was measured from the metabolite channel (Table 2) and results were calculated from peak areas using a linear VA (Sigma‐Aldrich, catalogue number 94770‐10G) standard curve ranging from 5 to 150 nM. Results were normalised to protein content, which was measured with Bradford protein assay (Bradford 1976).

### 2.3 Matrix‐Assisted Laser Desorption/Ionisation Mass Spectrometry

Samples for matrix‐assisted laser desorption/ionisation mass spectrometry (MALDI‐MSI) were collected by marking the orientation of the eye before enucleation, after which the eye was quickly embedded in polyvinylpyrrolidone (PVP, Sigma‐Aldrich, catalogue number 81420) and hydroxypropyl methylcellulose (HPMC, Methocel, 34693) medium (2.5 m/V‐% PVP, 7.5% HPMC in MilliQ water), and frozen on a liquid nitrogen‐cooled metal block and stored in −80°C. Sections were collected in the inferior–superior orientation at a thickness of 14 μm using Leica Cryostat CM3030 S and mounted onto indium tin oxide coated glass slides (Bruker Daltonics, catalogue number 8237001; Sigma‐Aldrich, 576352). DA detection with MALDI‐MSI was modified from previous brain‐tissue protocols (Shariatgorji et al. 2019; Shariatgorji et al. 2021). The samples were dried in a vacuum desiccator for 20–30 min. Next, the MALDI derivatization matrix with 1.8 mg/mL 4‐(anthracene‐9‐yl)‐2‐fluoro‐1‐methylpyridin‐1‐ium iodide salt (FMP‐10; Tag‐ON AB, catalogue number T1001) in 60% acetonitrile (Merck, 1.00030) in water (VWR, 1.15333) was sprayed over the retinal sections (TM‐sprayer, HTX‐Technologies, RRID:SCR_023732) with the following parameters: temperature 90°C, nitrogen gas pressure 6 bar, flow rate 80 μL/min for 50% acetonitrile pushing solvent, nozzle velocity 1100 mm/min, 20 passes, and 2 mm track spacing. MALDI MSI was performed using the timsTOF fleX instrument (Bruker Daltonics, RRID:SCR_023615) equipped with a smartbeam 3D laser. Data was collected in positive ion mode at a lateral resolution of 20 μm using the smart beam in beam scan mode with a scan range of 16 μm resulting in a laser field size of 20 μm. One burst of 160 laser shots was used to collect spectra from each position. Methods used were calibrated externally with red phosphorus and internally with FMP‐10 cluster ion (555.2231 m/z). Acquired data was analysed in SCiLS Lab (Version 13, Bruker Daltonics, RRID:SCR_014426). DA was detected as double derivatised at m/z 674.2803 m/z ±15 ppm across the retinal thickness from the inferior and superior retina, and ion intensity was exported as peak area normalised against total ion count. Only female WT and P23H tissues were used to limit the between‐animal variance of the method due to its low‐throughput nature, but the choice of utilised sex was arbitrary.

After, the sections were stained with haematoxylin and eosin. The samples were first rehydrated in > 99% and 94% ethanol (EtOH, 2 × 2 min each) and washed in MilliQ water for 20 s before a 7 min staining in Mayer's haematoxylin (Sigma‐Aldrich, catalogue number MHS32). After a 5 min wash under running tap water, samples were briefly (4 s) immersed in 1% hydrochloric acid in 94%, which was followed by a 10 min wash under tap water. Sections were then kept in 0.5% eosin Y (Sigma‐Aldrich, catalogue number E6003) alcoholic solution (94% with drops of glacial acetic acid) for 1 min and dehydrated in 94% and > 99% EtOH (2 × 2 min) before xylene clarification (2 × 5 min). Finally, the samples were covered with DPX Mountant (Sigma‐Aldrich, catalogue number 06522) and a cover slip. Samples were imaged with Apexview APX100 microscope (Evident Scientific).

### 2.4 Western Blotting

For western blotting (WB), the eye and the dissected retina were kept in 1XRIPA buffer (Millipore, catalogue number 20‐188) during collection. Frozen single retina samples were thawed on ice and incubated for 10 min in 60 μL of ice‐cold 1XRIPA buffer with 1% protease inhibitor (cOmplete Mini EDTA‐free Protease Inhibitor Cocktail, Roche, catalogue number 11836170001) before homogenisation with a handheld device. Samples from all tissue types were prepared similarly and the homogenisation volume for cortex, striatum and eye cup (remaining eye tissues without lens) samples was 150, 60, and 60 μL, respectively. After a further 15 min incubation on an ice bed, samples were centrifuged at +4°C for 20 min at 12000*g*. Sample volume was doubled with 2XLaemmli buffer (Bio‐Rad, catalogue number 1610737), and samples were boiled at +95°C for 5 min and cooled on ice for 5 min. Protein concentrations were equilibrated according to Pierce BCA protein assay (Thermo Scientific, catalogue number 23250). Gel electrophoresis was performed with Mini‐Protean TGX Stain‐free 4%–20% precast gels (Bio‐Rad, catalogue numbers 4568095, 4568096) and run at 200 V for 30 min. Gels were transferred to PVDF membranes (Bio‐Rad, catalogue number 1704157) with Trans‐Blot Turbo Transfer System (Bio‐Rad, RRID:SCR_023156) and blocked in 5% fat‐free milk (Valio) in tris‐buffered saline with 0.1% Tween 20 (TBST) for 1 h at room temperature. After three washes with TBST, membranes were incubated overnight in primary antibody (MS TH 1:2000, Cell Signalling Technology, RRID:AB_3677640; RB MAO‐A 1:2000, Invitrogen, catalogue number PA5‐79623; MS COMT 1:2000, BD Transduction Laboratories, RRID:AB_399391) in 5% milk TBST. The next day, the membranes were washed again with TBST, placed in secondary antibody (MS HRP 1:5000, Invitrogen, catalogue number A‐10668; MS IRDye 680 1:10000, Licor, catalogue number 925‐68070; RB HRP 1:10000, Cell Signalling Technologies, RRID:AB_2099233) in 5% milk TBST and incubated for 2 h at room temperature. Blot images were captured with Azure 600 (Azure Biosystems, RRID:SCR_023780) after washes with TBST and finally TBS. Chemiluminescence was induced with SuperSignal West Pico PLUS Chemiluminescent Substrate (Thermo Scientific, catalogue number 34577). Finally, the membranes were stained with Ponceau S (Thermo Scientific, catalogue number A40000278), and total protein staining was used to normalise the results. Images were analysed in ImageJ (version 1.54, RRID:SCR_003070) with the lane plot tool (Schneider et al. 2012). Uncropped western blot images are presented in Figures S2 and S3.

### 2.5 Quantitative Polymerase Chain Reaction

For real‐time quantitative polymerase chain reaction (qPCR), RNA extraction was first performed with RNeasy Mini Kit (Qiagen, catalogue number 74104). Prior to the extraction, the samples were soaked overnight in RNAlater‐ICE solution (Ambion, catalogue number AM7030) at −20°C, and RNA extraction included on‐column DNase digestion with RNase‐Free DNase Set (Qiagen, catalogue number 79256). RNA quantity and quality were assessed with Denovix DS‐11, and RNA concentrations were adjusted with water for molecular biology (BioNordika, catalogue number BN‐51100). Complementary DNA (cDNA) was synthesised with iScript cDNA Synthesis Kit (Bio‐Rad, catalogue number 1708891), and the final product was diluted 10× with water. For qPCR, a solution with PowerUp SYBR Green Master Mix (5 μL per sample, Applied Biosystems, catalogue number A25741), forward and reverse primers (Table 3, 0.5 μL per sample per primer, Merck) and water (3 μL per sample) was prepared and 9 μL of it was added per well to a 96‐well PCR plate (VWR, 732‐2389). Each sample was analysed in triplicate with a sample volume of 1 μL per well. The plate was covered with a plastic membrane (VWR, catalogue number 89087‐690) and centrifuged to remove air bubbles. The PCR reaction was initiated and measured with QuantStudio 5 qPCR device (Applied Biosystems, RRID:SCR_020240; Stage 1: 2 min at 50°C, 2 min at 95°C; Stage 2: 15 s at 95°C, 30 s at 60°C, repeated 40 times). Results were analysed in Microsoft Excel (RRID:SCR_016137) and calculated with the ΔΔCt method using succinate dehydrogenase complex flavoprotein subunit A (*Sdha*) and (*Gapdh*) expression for normalisation.

**TABLE 3:** Primer sequences used in quantitative polymerase chain reaction.

Gene	Forward primer	Reverse primer
*Th*	TGCACACAGTACATCCGTCATGC	GCAAATGTGCGGTCAGCCAACA
*Aadc*	AGCTGACTATCTGGATGGCAT	ACCCCTGGCATGATTATCTTCT
*Maoa*	CCCTGTGGTTCTTGTGGTATG	GCTCAGCTTCACTTTATCCCC
*Maob*	GATCTCTCGTGTGCCTTTGG	GCTTCCTCTCCTTCGATAACC
*Comt*	CTGGGGCTTGGTGGCTATTG	CTTTTGCGTCACCCACGTTC
*Pde6a*	CAGCAACTACCACGATGTGAA	GTGGACATTGAAGAGCCTAGTG
*Pcp2*	ATGGACGACCAGCGTGTAAC	CCTTGGGGCCGATAGGTTG
*Gfap*	CGGAGACGCATCACCTCTG	AGGGAGTGGAGGAGTCATTCG
*Sdha*	GCTCCTGCCTCTGTGGTTGA	AGCAACACCGATGAGCCTG
*Gapdh*	TGACGTGCCGCCTGGAGAAA	AGTGTAGCCCAAGATGCCCTTCAG

Abbreviations: *Aadc* = aromatic L‐amino acid decarboxylase, *Comt* = catechol‐O‐methyltransferase, *Gapdh* = glyceraldehyde 3‐phosphate dehydrogenase, *Gfap* = glial fibrillary acidic protein, *Maoa* = monoamine oxidase A, *Maob* = monoamine oxidase B, *Pcp2* = Purkinje cell protein 2, *Pde6a* = phosphodiesterase 6A, *Sdha* = succinate dehydrogenase complex flavoprotein subunit A, *Th* = tyrosine hydroxylase.

### 2.6 Immunohistochemistry

The eyes were enucleated and the corneas were cut before fixing the remaining eye in 4% (V/V) paraformaldehyde (Electron Microscopy Sciences, catalogue number 15710) for 1 h, after which the iris and the lens were removed. The samples were then dehydrated in sucrose solutions (10%/20%/30% (m/V, Sigma‐Aldrich, catalogue number S9378) in 1XPBS), equilibrated in the embedding medium overnight, and embedded the next day in the same 1:2 20%‐sucrose/Frozen Section Media (Leica Biosystems, catalogue number 3801480). The samples were frozen on a liquid nitrogen‐cooled metal block and stored at −80°C before sectioning. Eye cup sections (10 μm) were collected with Leica Cryostat CM3030 S onto microscope slides (Epredia, catalogue number J1800AMNZ).

For antibody staining, the sections were first surrounded with a hydrophobic barrier (ImmEdge, catalogue number H‐4000), and the embedding medium was removed with a 10 min wash in 1XPBS. Next, sections were incubated for 30 min in a 10% (V/V) normal donkey serum (NDS, Biowest, S2170) blocking solution before an overnight primary antibody (RB TH 1:500, Proteintech, RRID:AB_2716568; RB MAO‐A 1:500, Invitrogen, catalogue number PA5‐79623; MS COMT 1:500, BD Transduction Laboratories, RRID:AB_399391) incubation at 4°C in PBST (0.1% (V/V) Tween 20) with 1% NDS. Next day, the samples were washed three times with PBST for 5 min and incubated for 2 h with secondary antibodies (MS 488 1:1000, Abcam, RRID:AB_2732856; RB 647 1:1000, Abcam, RRID:AB_2752244) at room temperature in the dark. The sections were then washed three times with PBST, and twice with PBS before being covered with a 4′,6‐diamidino‐2‐phenylindole (DAPI)‐containing mounting medium (Abcam, catalogue number AB104139). Sections were imaged with Olympus APX100 fluorescence microscope (Evident Scientific); TH with 40× magnification and MAO‐A and COMT with 20×.

### 2.7 Bulk RNA Sequencing

RNA extraction from P12 and P18 P23H and WT mouse retinas was performed as described above. Library preparation and sequencing were conducted by Novogene using the Plant and Animal Eukaryotic mRNA (WOBI) package. Briefly, poly(A) + mRNA was enriched from total RNA using oligo‐dT magnetic beads, followed by fragmentation and first‐strand cDNA synthesis using random hexamer primers. Libraries were generated through end repair, A‐tailing, adapter ligation, size selection, amplification, and purification, and sequenced on an Illumina platform. Raw reads were subjected to quality filtering by the provider, including removal of adapter‐containing reads, reads containing > 10% undetermined bases, and reads in which > 50% of bases had quality scores ≤ 5. Sequencing quality was high across samples (Q20 > 99%, error rate ~0.01%).

The quality of raw RNA‐seq reads was assessed using FastQC (v0.11.9, RRID:SCR_014583, Andrews 2010) to evaluate sequence quality, GC content, and potential adapter contamination. Initial preprocessing, alignment, and read counting steps up to the generation of sample‐wise count matrices were carried out using the Puhti high‐performance computing system operated by CSC—IT Center for Science, Finland. Reads were aligned to the *Mus musculus* GRCm38.p6 genome assembly using STAR (v2.7.11b, RRID:SCR_004463, Dobin et al. 2013) with the GRCm38.p6 genome FASTA file (GRCm38.p6.genome.fa) and the corresponding GENCODE (RRID:SCR_014966) mouse release M25 annotation file (gencode.vM25.annotation.gtf). GENCODE M25 is based on the GRCm38.p6 mouse genome assembly (https://www.gencodegenes.org/mouse/releases.html?utm_source=chatgpt.com). Feature counting was performed using HTSeq (v2.0, Putri et al. 2022) to generate the read count matrix for each sample. Downstream normalisation and differential expression analysis were conducted in R (v4.5.2) using DESeq2 (v1.50.2, RRID:SCR_015687, Love et al. 2014). The data have been deposited in NCBI GEO (GEO accession number GSE334684). In addition, publicly available P23H and WT retinal RNA‐seq datasets collected at P30 (GSE152474) and P90 (GSE156533) were downloaded from NCBI GEO and reanalysed using the same bioinformatics pipeline. These datasets originate from prior work by the senior author (Leinonen et al. 2020). Sample collection, handling, and sequencing procedures were comparable between newly generated and publicly available cohorts.

Each genotype comparison was performed within a single age. Differential expression was assessed in DESeq2, with *p*‐values adjusted for multiple testing to control the false discovery rate (FDR). Genes were considered differentially expressed (DEGs) at adjusted *p* < 0.05 and an absolute log2 fold‐change greater than 0.585 (1.5‐fold). Candidate catecholaminergic genes, pre‐specified on the basis of the biochemical findings described above rather than identified from the genome‐wide screen, were examined separately using adjusted *p* < 0.05 as a criterion alone, without an effect‐size filter.

### 2.8 Retinal Layer Separation

Retinal layer separation (ReLayS) samples were collected according to previous literature (Todorova et al. 2022). Briefly, the retina was flat‐mounted on a microscope slide with the ganglion cell layer facing up, and a 0.5 × 0.5 cm piece of Durapore membrane (Millipore, catalogue number DVPP04700) was placed on top to adhere the retina to the membrane. After freezing the membrane on a dry ice‐cooled metal block, the membrane was thawed at room temperature on a moistened piece of Whatman filter paper (catalogue number WHA1001045), and a new piece of membrane was placed on top of the retina and peeled to separate the photoreceptor layer. The freezing and thawing procedure was repeated to separate the outer nuclear layer from the remaining inner retina. Samples for qPCR were snap‐frozen, and samples intended for histology were fixed for 24 h at 4°C and then transferred to 70% EtOH until paraffin processing. For paraffin embedding, samples were placed in tissue processing cassettes (Macrostar VI, VWR, catalogue number 720‐2178), and a drop of HistoGel (Epredia, HG‐4000‐012) was pipetted onto each sample before being wrapped in Bio‐Wrap (Leica, 3 801 090). Samples were dehydrated through increasing concentrations of EtOH, cleared with xylene and infiltrated in paraffin overnight, then embedded in paraffin moulds the following day. Samples were cut into 5 μm‐thick sections with a microtome (Thermo Scientific, HM355S, RRID:SCR_026146) and dried overnight at 37°C. Haematoxylin and eosin staining was performed similarly to as described above with additional deparaffinisation steps (2 × 5 min) at the start, and samples were imaged with the Apexview APX100 microscope (Evident Scientific).

### 2.9 Statistical Analysis

Results were analysed in GraphPad Prism (version 10.6, RRID:SCR_002798) and are presented as mean ± SD. Normality of the data was determined with Shapiro–Wilk test (Supporting Information S1), and statistical significance was assessed accordingly with parametric two‐tailed unpaired or Welch's *t*‐test, ordinary or Brown‐Forsythe one‐way analysis of variance (ANOVA) with Tukey's or Dunnett's T3 post hoc test, two‐way ANOVA with Šídák's multiple comparisons test, or the nonparametric Mann–Whitney test (Supporting Information S1). For UHPLC datasets consisting of WT and RP mouse comparisons in multiple age points or light conditions, multiple *t*‐tests or Mann–Whitney tests were performed with Holm‐Šidák correction for multiple comparisons. No priori sample size calculation was performed; sample sizes were based on previous studies of similar nature (Park et al. 2013; Pérez‐Fernández et al. 2017, 2019) and sample availability. Possible statistical outliers were identified with Rout method (*Q* = 1%, Supporting Information S1). The level of statistical significance was set to *p* < 0.05.

## 3 Results

### 3.1 Increased Retinal Dopamine Content in Developing and Adult RP Retinas

**FIGURE 1:**
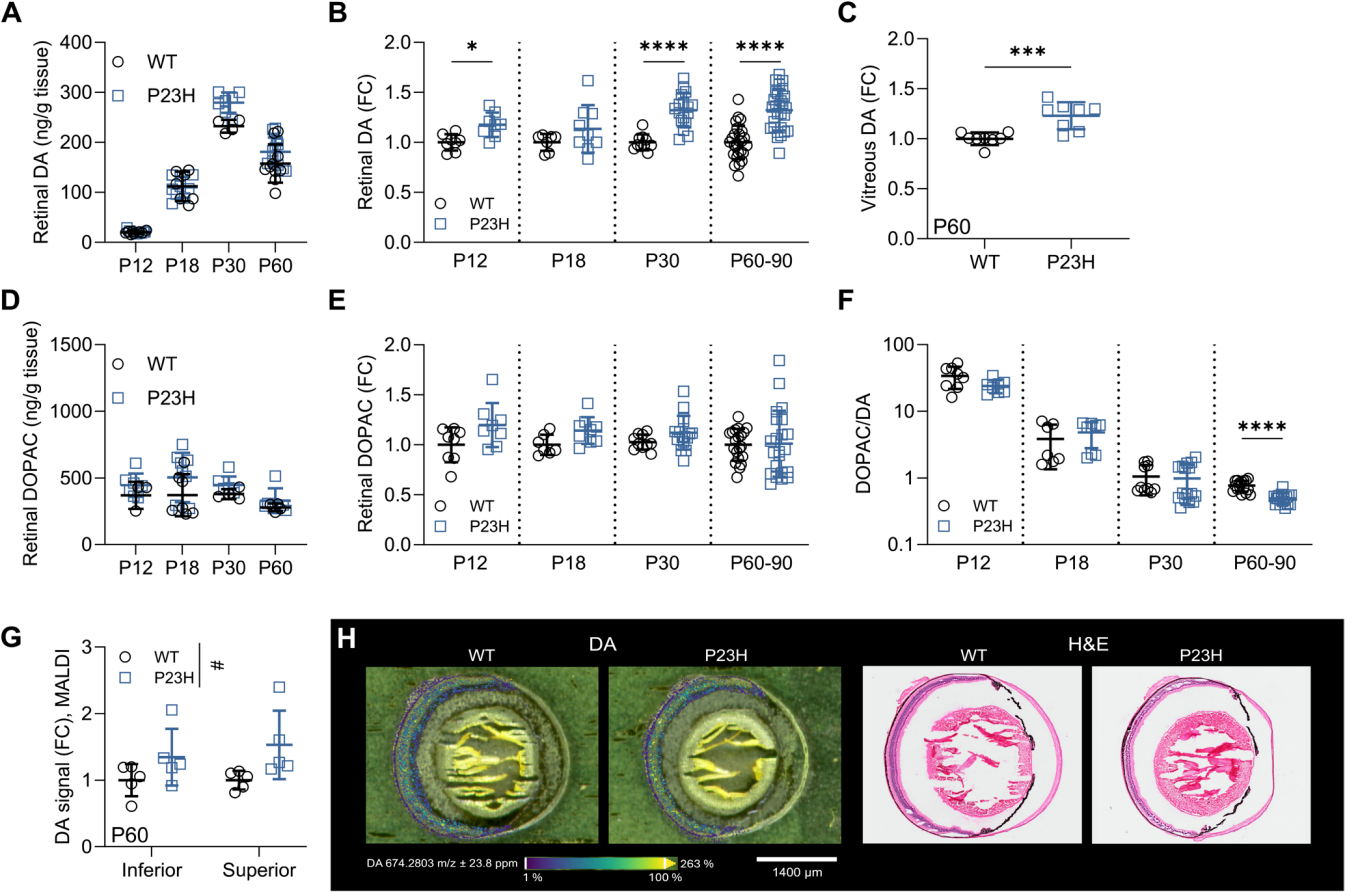
Elevation of retinal dopamine (DA) content in P23H rhodopsin‐mutant mice representing autosomal dominant retinitis pigmentosa. (A) Retinal DA (ng/g tissue) measured with ultra‐high‐performance liquid chromatography across ages in wild‐type (WT) and P23H mice at P12 (WT *n* = 8, P23H *n* = 8), P18 (WT *n* = 7, P23H *n* = 9), P30 (WT *n* = 4, P23H *n* = 7) and P60 (WT *n* = 10, P23H *n* = 12) with data from comparable batches. (B) DA in P23H retinas in relation to healthy age‐matched WT group (P12; WT *n* = 8, P23H *n* = 8; P18 WT *n* = 7, P23H *n* = 9; P30 WT *n* = 10, P23H *n* = 17; P60‐90 WT *n* = 26, P23H *n* = 28), pooled from independent batches. (C) DA measured from vitreous, *n* = 8 per group. (D) Retinal 3,4‐dihydroxyphenylacetic acid (DOPAC, ng/g tissue) across ages in WT and P23H mice at P12 (WT *n* = 3, P23H *n* = 7), P18 (WT *n* = 7, P23H *n* = 9), P30 (WT *n* = 4, P23H *n* = 7) and P60 (WT *n* = 4, P23H *n* = 6) with data from comparable batches. (E) DOPAC in P23H retinas relative to the healthy age‐matched WT group (P12; WT *n* = 8, P23H *n* = 8; P18 WT *n* = 7, P23H *n* = 9; P30 WT *n* = 10, P23H *n* = 16; P60‐90 WT *n* = 20, P23H *n* = 22), pooled from multiple experiments. (F) Numerical DOPAC/DA ratio across ages in WT and P23H mice (P12; WT *n* = 8, P23H *n* = 8; P18 WT *n* = 7, P23H *n* = 9; P30 WT *n* = 10, P23H *n* = 16; P60‐90 WT *n* = 16, P23H *n* = 16). (G) Analysis of DA signal peak area as measured with matrix‐assisted laser desorption/ionisation mass spectrometry (MALDI‐MSI) from retinas of WT and P23H mice at P60, *n* = 5 per group. (H) Example images from DA detection with MALDI‐MSI (left) together with the corresponding haematoxylin and eosin (H&E) staining image (right). Fold‐change (FC) in graphs B, C, E, and G refers to ratio change compared to WT mean value. Data are presented as mean ± SD, and hashtag symbol in panel G represents significant two‐way ANOVA main effect between genotypes. Asterisks indicate Mann–Whitney test (panel B, C, F; B and F adjusted for multiple comparisons with Holm‐Šidák method) results respectively: #/* = *p* < 0.05, ** = *p* < 0.01, *** = *p* < 0.001, **** = *p* < 0.0001.

DA and DOPAC contents in P23H and WT littermate retinas were assayed with UHPLC at various time points (P12–90, Figure 1). At P12, before eye opening, DA levels were the lowest, and between P12 and P30 mean DA levels increased approximately 14‐fold (Figure 1A). DA amounts decreased about 30% between P30 and P60 as the mice reached adulthood. Since DA levels change rapidly across postnatal development and because mice at different ages represent biologically distinct disease stages, statistical comparisons of UHPLC analytes between WT and RP mice were performed at each time point using multiple *t*‐tests or Mann–Whitney tests with Holm‐Šidák correction for multiple comparisons (Figure 1B). We observed increased DA levels in P23H retinas already at P12 (Mann–Whitney *U* = 8, adjusted *p* = 0.0207). After eye opening at P18, there was no statistically significant difference between WT and P23H. However, retinal DA was markedly higher in juvenile (P30; Mann–Whitney *U* = 5, adjusted *p* < 0.0001) and adult (P60–90) P23H samples (Mann–Whitney *U* = 80, adjusted *p* < 0.0001) in comparison to the healthy WT tissue. To account for batch‐to‐batch variation, analyte values were normalised to the WT mean within each experiment before statistical comparison. Results from both sexes were included and analysed together, but the data was evaluated for possible sex differences prior to the analyses (Figure S1, Supporting Information S1).

Next, we estimated DA release and turnover using vitreous DA concentrations at P60 (Figure 1C) and retinal levels of the DA metabolite DOPAC measured across P12 to P90 (Figure 1D,E). Analysis of vitreous samples revealed an approximately 30% increase in DA levels in P23H mice compared with WT controls (Mann–Whitney test *U* = 2, *p* = 0.0006, Figure 1C). Retinal DOPAC amounts were generally higher in pre‐weaned P23H mice (Figure 1E), although statistical significance was not reached. No differences were evident in the older groups. The DOPAC/DA ratio, commonly used as an index of DA metabolism and turnover (Witkovsky 2004), decreased after eye opening and development (Figure 1F), and in adult mice the ratio was lower in P23H retinas compared with WT (Mann–Whitney *U* = 9, adjusted *p* < 0.0001).

Finally, retinal DA was additionally assessed with MALDI‐MSI from female WT and P23H samples at a single time point (P60). Analysis of signal peak area corroborated UHPLC results by revealing an increase in DA signal in P23H retinas (*F*(1,16) = 7.354, *p* = 0.0154, Figure 1G). Moreover, the results display the diffuse nature of this retinal catecholamine (Figure 1H).

**FIGURE 2:**
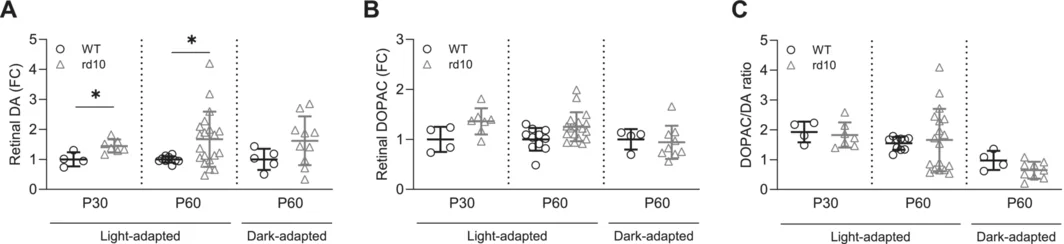
Retinal dopamine (DA) is elevated in *Pde6b*‐mutant rd10 mice representing autosomal recessive retinitis pigmentosa. (A) Ultra‐high performance liquid chromatography measurements of retinal DA from light‐adapted wild‐type (WT) and rd10 retinas collected at P30 (WT *n* = 4, rd10 *n* = 7) and P60 (WT *n* = 10, rd10 *n* = 20), and from dark‐adapted mice collected at P60 (WT *n* = 5, rd10 *n* = 10). (B) 3,4‐dihydroxyphenylacetic acid (DOPAC) content in light‐adapted WT and rd10 retinas collected at P30 (WT *n* = 4, rd10 *n* = 7) and P60 (WT *n* = 11, rd10 *n* = 18), and from dark‐adapted mice collected at P60 (WT *n* = 4, rd10 *n* = 9). (C) DOPAC/DA ratio in WT and rd10 mice (P30 WT *n* = 4, rd10 *n* = 7; light‐adapted P60 WT *n* = 10, rd10 *n* = 18; dark‐adapted WT *n* = 4, rd10 *n* = 9). Mice were housed in the dark throughout their lives. However, sample collection was performed in either a light‐ or dark‐adapted state. Light adaptation was carried out by exposing the animals to normal laboratory light for 15 min before sample collection. Dark‐adapted mice were euthanized in the dark room under dim red light. Fold‐change (FC) in graphs A and B refers to ratio change compared to WT mean value. Data are presented as mean ± SD, and hashtags and asterisks indicate significant statistical Welch's *t*‐test (panel A, adjusted for multiple comparisons with Holm‐Šidák method), respectively: * = *p* < 0.05.

To increase the translational relevance of the results to RP rather than studying mutation‐specific effects, we also assessed DA and DOPAC levels in rd10 mouse retinas collected at P30 and P60 in light‐adapted and dark‐adapted states (Figure 2). Similar to the findings from P23H mice, there was a significant increase in DA content in light‐adapted rd10 retinas at P30 (unpaired *t*‐test with Welch correction *t* = 3.085, df = 6, adjusted *p* = 0.0426 after Holm‐Sídák correction, Figure 2A) and P60 (unpaired *t*‐test with Welch correction *t* = 3.212, df = 20, adjusted *p* = 0.0131). In contrast, in the dark‐adapted state at P60 there was only a non‐significant trend towards elevated retinal DA levels between the rd10 and control WT samples (unpaired *t*‐test with Welch correction *t* = 2.054, df = 12.92, adjusted *p* = 0.0608). Furthermore, there was some increase in DOPAC levels in light‐adapted rd10 mice at P60 nearing statistical significance (Mann–Whitney test *U* = 47, adjusted *p* = 0.0553, Figure 2B). Retinal DOPAC/DA ratios were similar between genotypes (Figure 2C).

### 3.2 Catecholamine‐o‐Methyltransferase Expression Is Comparable Between the Mouse Retina, Striatum, and Brain Cortex

**FIGURE 3:**
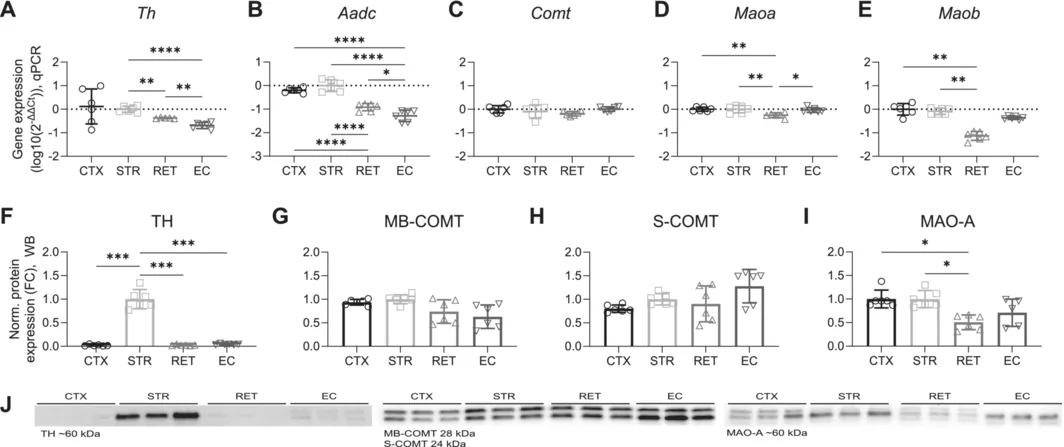
Catechol‐O‐methyltransferase expression is similar in the retina and dopaminergic brain areas, whereas retinal monoamine oxidase expression is low. Dopamine‐synthetising (TH and AADC) and metabolising (COMT and MAOs) enzymes were analysed with quantitative polymerase chain reaction (qPCR, panels A–E) and western blotting (WB, panels F–J) from the cortex (CTX) and striatum (STR) of the brain, as well as from the retina (RET) and eye cup (EC) of adult (~P60) wild‐type mice. Top panels show normalised gene expressions of (A) tyrosine hydroxylase (*Th*), (B) aromatic‐L‐amino‐acid decarboxylase (*Aadc*), (C) catechol‐o‐methyltransferase (*Comt*), and (D, E) monoamine oxidases A and B (*Maoa*, *Maob*) as measured with qPCR, log10(2^−∆∆Ct^). Panels F‐I show normalised expression of (F) TH, (G) membrane‐bound (MB‐) COMT, (H) soluble (S‐) COMT, and (I) MAO‐A proteins as measured with WB. FC indicates fold‐change compared to the mean value of STR samples. The bottom panel J displays representative immunoblots. The corresponding uncropped blot images are provided in Figure S2. Group size *n* = 6, except for panel A where RET *n* = 5 and panel I where EC *n* = 5. Data are presented as mean ± SD, and asterisks present significant statistical Tukey's (panels B, D), Dunnett's T3 (panels A, F) or Dunn's (panels E, I) test results, respectively: * = *p* < 0.05, ** = *p* < 0.01, *** = *p* < 0.001, **** = *p* < 0.0001.

Because the mechanisms responsible for regulating dopaminergic activity and turnover in the retina are largely unknown, we next evaluated the expression of enzymes involved in DA synthesis and metabolism in healthy WT mouse retinas. Expression levels were compared with those in the striatum and cortex of the brain, where dopaminergic transmission is better characterised, and with the eye cup (remaining ocular tissue after retinal extraction, our sample included the cornea, iris, and sclera), to place retinal expression levels into a physiological context. Gene and protein expression were quantified using qPCR and WB (Figure 3). Expression of the DA‐synthetising enzymes was generally higher in the brain tissues. *Th* was significantly lower in the eye cup than in the striatum or retina (F*(3.642) = 5.564, Brown‐Forsythe ANOVA test *p* = 0.0397, Dunnett's T3 multiple comparison test between eye cup and striatum *p* < 0.0001, eye cup and retina *p* = 0.0099, Figure 3A). In contrast, *Aadc* was expressed at very low (C_t_ values > 30) levels in the eye tissues in comparison to both brain regions (*F*(3,20) = 56.08, main ANOVA effect *p* < 0.0001, Tukey's multiple comparisons to cortex and striatum *p* < 0.0001, Figure 3B). Notably, however, *Aadc* mRNA levels were higher in the retina than in the eye cup (Tukey's *p* = 0.0194). At the protein level, the striatum distinctly displayed the strongest TH signal (F*(3,5.122) = 133.2, Brown‐Forsythe ANOVA test *p* < 0.0001, Dunnett's T3 multiple comparison test *p* = 0.0004 between striatum and other tissues, Figure 3F, Figure S2).

In terms of metabolising enzymes, the gene expression of *Comt*, as well as membrane‐bound (MB‐) and soluble (S‐) COMT protein levels, were largely comparable between the retina, eye cup, striatum and cortex samples (Figure 3C,G,H,J), suggesting a broadly similar capacity for COMT‐mediated catecholamine methylation across the tissues assayed. In contrast, the expression of *Maoa* and *Maob* showed pronounced tissue‐specific differences. *Maoa* transcript levels were significantly lower in the retina compared with the other tissues (*F*(3,20) = 6.745, main ANOVA effect *p* = 0.0025, Tukey's multiple comparisons between retina and cortex *p* = 0.0058, striatum *p* = 0.0055, and eye cup *p* = 0.0159, Figure 3D), and *Maob* mRNA expression was even lower compared with the brain tissues (Kruskal‐Wallis test statistic = 17.90, *p* = 0.0005; Dunn's multiple comparisons cortex vs. retina *p* = 0.001, striatum vs. retina *p* = 0.0042, Figure 3E). Overall, *Maob* was minimally expressed (mean C_t_ ~ 30). In line with gene expression, MAO‐A protein expression was lower in the retina compared with the brain tissues (Kruskal‐Wallis test statistic = 12.24, *p* = 0.0066; Dunn's multiple comparisons between retina and cortex *p* = 0.0228, and striatum *p* = 0.0151, Figure 3I,J). Based on these results, COMT may play a more significant role in DA metabolism in the retina than MAOs. TH and COMT were used as the main enzyme targets in further evaluations of DA synthesis and metabolism in adult RP retinas.

### 3.3 Catechol‐O‐Methyltransferase‐Mediated DA Metabolism Increases in Adult RP Retinas

**FIGURE 4:**
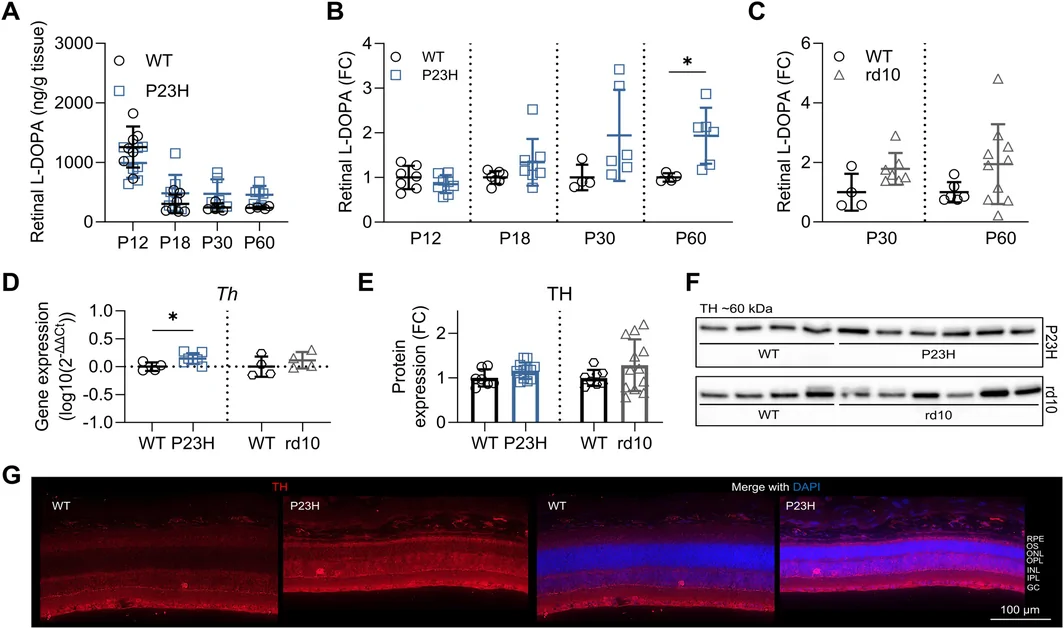
Analysis of dopamine synthesis markers in retinitis pigmentosa retinas compared with those of healthy mice. (A) Retinal l‐3,4‐dihydroxyphenylalanine (L‐DOPA, ng/g tissue) content in wild‐type (WT) and P23H mice at P12 (WT = 7, P23H = 8), P18 (WT = 7, P23H = 8), P30 (WT = 4, P23H = 6) and P60 (WT = 4, P23H = 6). (B) L‐DOPA in P23H retinas in relation to healthy age‐matched WT group (P12 WT *n* = 7, P23H *n* = 8; P18 WT *n* = 7, P23H *n* = 8; P30 WT *n* = 4, P23H *n* = 6; P60 WT *n* = 4, P23H *n* = 6). (C) L‐DOPA content in light‐adapted rd10 mouse retinas at P30 and P60 in relation to WT group (P30 WT *n* = 4, rd10 *n* = 7; P60 WT *n* = 6, rd10 *n* = 10). (D) Normalised expression of tyrosine hydroxylase (*Th*) mRNA in light‐adapted WT (*n* = 4 in both experiments), P23H (*n* = 7) and rd10 (*n* = 4) mouse retinas collected at ~P60, log10(2^−∆∆Ct^). (E) Normalised expression of TH protein as measured with western blotting from light‐adapted adult P23H (P90, *n* = 12), rd10 (P60, *n* = 12) and WT retinas (P90 *n* = 7, P60 *n* = 8). (F) Representative TH immunoblots. The corresponding uncropped blot images are provided in Figure S3. (G) Representative fluorescence microscopy images from WT and P23H retinal cryosections stained with TH antibody (red) and 4′,6‐diamidino‐2‐phenylindole (DAPI, blue). Three biological replicates per group were assayed with similar results, as shown in Figure S4A. Fold‐change (FC) in graphs B, C, D and E refers to ratio change compared to WT mean value. Numerical data is presented as mean ± SD. The asterisks denote significant Mann–Whitney test (panel B, adjusted with Holm‐Šidák correction) or *t*‐test (panel D) results, respectively: #/* = *p* < 0.05.

**FIGURE 5:**
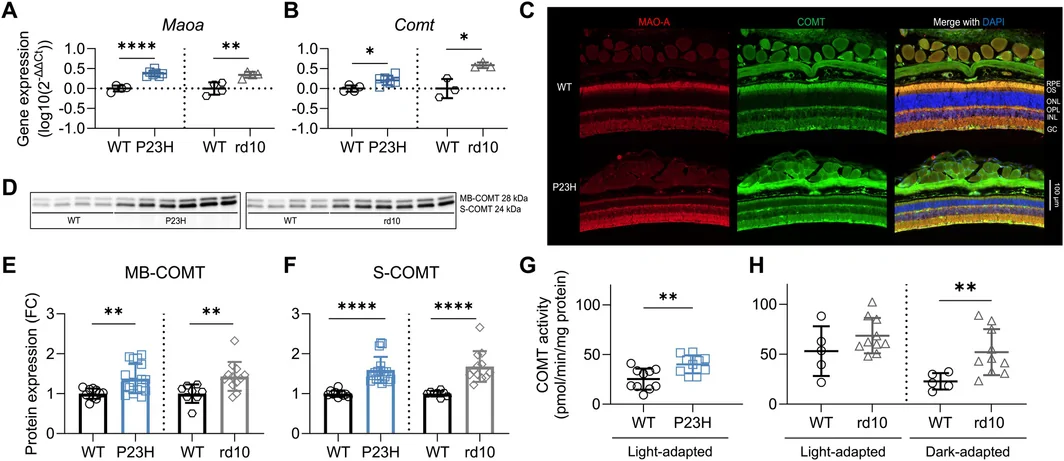
Upregulated retinal catechol‐O‐methyltransferase expression in mouse retinitis pigmentosa. Normalised mRNA expression of (A) monoamine oxidase A (*Maoa*) and (B) catechol‐O‐methyltransferase (*Comt*) measured from light‐adapted adult (P60) wild‐type (WT, *n* = 3–4), P23H (*n* = 6–7) and rd10 retinas (*n* = 3–4), log10(2^−∆∆Ct^). (C) Representative fluorescence microscopy images from adult WT and P23H retinal cryosections stained with MAO‐A antibody (red), COMT antibody (green) and 4′,6‐diamidino‐2‐phenylindole (DAPI, blue). Three biological replicates per group were assayed with similar results, as shown in Figure S4B. (D) Representative COMT immunoblots. The corresponding uncropped blot images are provided in Figure S3. Statistical analysis of (E) membrane‐bound (MB) COMT, and (F) soluble (S) COMT measured from light‐adapted adult P23H (P90, *n* = 17), rd10 (P60, *n* = 12) and WT mice (P90 *n* = 10, P60 *n* = 8), fold‐change (FC) to WT. (G) COMT enzyme activity assay results from light‐adapted adult P23H (P90, *n* = 10), and (H) rd10 (P60, *n* = 10 in both adaptation states) retinas in comparison to WT mouse retinas (P23H experiment *n* = 10, rd10 experiments *n* = 5), pmol/min/mg of protein. Data are presented as mean ± SD, and asterisks indicate significant statistical *t*‐test (panels A, B, rd10 E and F, G, H, H adjusted for multiple comparisons with Holm‐Šidák method) or Mann–Whitney test results (P23H panel E and F), respectively: * = *p* < 0.05, ** = *p* < 0.01, *** = *p* < 0.001, **** = *p* < 0.0001.

Next, we proceeded to assess parameters related to DA synthesis (Figure 4) and metabolism in RP (Figure 5). As with DA, age impacted retinal L‐DOPA (Figure 4A), as just prior to the eye opening at P12 L‐DOPA levels were over 2‐fold higher than 6 days later at P18, which represents a time point when mouse eyes have been open for a few days. L‐DOPA amounts appeared slightly higher in P23H retinas after P18, although statistical significance was reached at P60 only (Mann–Whitney *U* = 0, adjusted *p* = 0.0376 after Holm‐Sídák correction, Figure 4B). In light‐adapted rd10 mouse retinas at P30 or P60, L‐DOPA levels did not significantly differ from the corresponding WT samples (Figure 4C).

DA synthesis was further evaluated in WT and RP retinas (P23H P60–90, rd10 P60) using qPCR and WB assessment of *Th*/TH. The transcript levels were upregulated in P23H retinas in contrast to WT (unpaired *t*‐test *t* = 2.574, df = 9, *p* = 0.03, Figure 4D), but at the protein‐expression level there was no significant difference (Figure 4E, Figure S3). There was no alteration in either mRNA or protein expression in rd10 mouse retinas compared to WT (Figure 4E,F). Retinal TH localisation was visualised by the means of immunohistochemistry staining and fluorescence microscopy in adult WT and P23H cryosections (Figure 4G). In line with literature (Brecha et al. 1984), TH signal was strongest in the interface between the inner nuclear and plexiform layers, with sparse dopaminergic amacrine cell bodies visible. This was further supported by WT qPCR results from ReLayS samples (Figures S4 and S5).

With regard to DA metabolism, both *Maoa* and *Comt* transcript levels were elevated in adult (P60) P23H and rd10 retinas compared with their healthy WT counterparts (*Maoa* unpaired *t*‐test P23H *t* = 8.347, df = 9, *p* < 0.0001; rd10 *t* = 4.036, df = 6, *p* = 0.0068; *Comt* unpaired *t*‐test P23H *t* = 2.987, df = 8, *p* = 0.0174; rd10 *t* = 4.038, df = 4, *p* = 0.0156; Figure 5A,B). Immunohistochemical staining of WT and P23H samples showed that both metabolising enzymes are expressed in the inner retina, with COMT signal being particularly strong at the retinal ganglion cell layer (Figure 5C). This finding was corroborated by the analysis of mRNA transcript levels after ReLayS (Figures S4 and S5). Based on the data from the comparisons between brain and eye tissues (Figure 3), we focused our further investigations on the COMT enzyme. COMT protein expression was evaluated with immunoblotting that also allowed the separate analysis of MB‐ and S‐COMT separately (Figure 5D–F). Both forms of this metabolising enzyme were upregulated by the disease in P23H (P90; MB‐COMT Mann–Whitney test *U* = 29, *p* = 0.0039; S‐COMT Mann–Whitney test *U* = 0, *p* < 0.0001) and rd10 retinas (P60; MB‐COMT unpaired *t*‐test *t* = 2.974, df = 18, *p* = 0.0081; S‐COMT Welch's *t*‐test *t* = 5.984, df = 12.08, *p* < 0.0001). Finally, COMT enzymatic activity was assayed with a UHPLC‐based method from 3‐month‐old P23H and 2‐month‐old rd10 retinas together with age‐matched WT samples (Figure 5G,H). Samples from both light‐ and dark‐adapted animals were collected for the rd10 experiments. In line with COMT mRNA transcript and protein levels, retinal enzyme activity was additionally elevated in P23H (P90; unpaired *t*‐test *t* = 3.362, df = 18, *p* = 0.0035, Figure 5G) and dark‐adapted rd10 mice (P60; unpaired *t*‐test with Welch correction *t* = 3.581, df = 12.37, adjusted *p* = 0.0072 after Holm‐Sídák correction, Figure 5H). However, the comparison between light‐adapted rd10 and corresponding WT retinas did not reveal statistically significant results. Nevertheless, the upregulation of COMT‐mediated metabolism is evident in RP mouse retinas.

### 3.4 RNA Sequencing Indicates Widespread Alterations in the Catecholaminergic System in P23H Retinas

**FIGURE 6:**
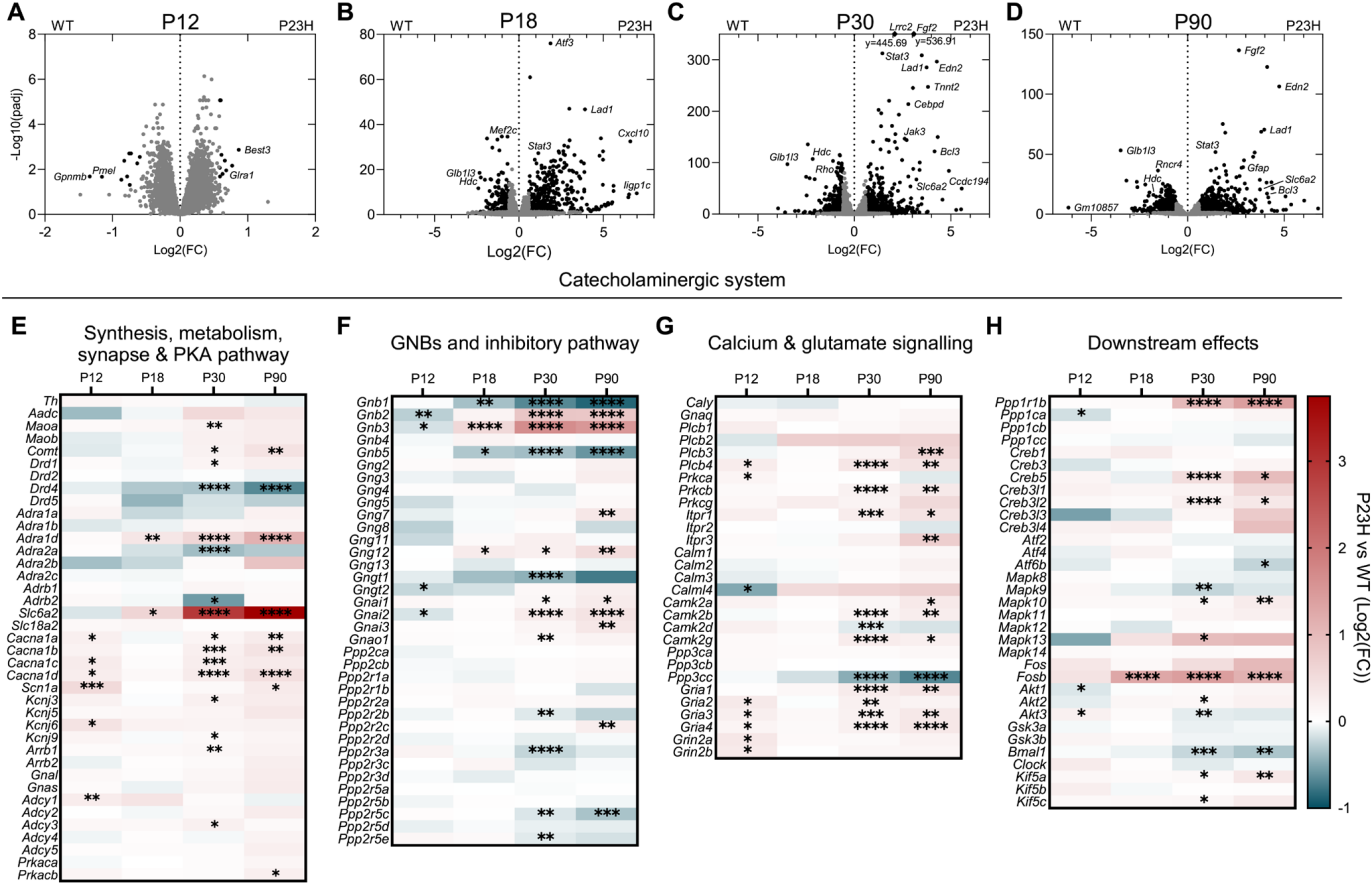
Widespread alterations in the retinal catecholaminergic transcriptome of P23H mice. Volcano plots of identified genes in wild‐type (WT) and P23H retinas collected at (A) P12 (WT *n* = 5, P23H *n* = 6), (B) P18 (WT *n* = 5, P23H *n* = 6), (C) P30 (WT *n* = 7, P23H *n* = 7) and (D) P90 (WT *n* = 3, P23H *n* = 4) with differentially expressed genes (log2(fold‐change, FC) > 0.585, adjusted *p*‐value < 0.05) marked in black. Several transcripts associated with the catecholaminergic system were evaluated separately without log2(FC) limit, and heatmaps represent alterations in genes related to (E) synthesis, metabolism, synapse and protein kinase A (PKA) pathway, (F) guanine nucleotide‐binding proteins (GNBs) and inhibitory pathway, (G) calcium and glutamate signalling, and (H) downstream effects. Asterisks present significant adjusted *p*‐values, respectively: * = *p* < 0.05, ** = *p* < 0.01, *** = *p* < 0.001, **** = *p* < 0.0001.

Bulk retinal RNA sequencing data from P23H and littermate WT mice collected at P12, P18, P30 and P90 were analysed to obtain a comprehensive systems‐level view of catecholaminergic pathway alterations. Data from P12 and P18 mice were generated in this study, whereas P30 and P90 datasets were obtained from previously published, open‐access RNA‐seq data (Leinonen et al. 2020). The onset and progression of retinal degeneration was evident in the data, as the number of DEGs increased more than 30‐fold from P12 to P18, and again over two‐fold from P18 to P30 and P90 (P12: 20 DEGs out of 34 577 identifications; P18: 624 DEGs out of 34 098; P30: 1304 DEGs out of 35 505; P90: 1407 DEGs out of 31 785; log2(fold‐change) > 0.585, adjusted *p*‐value < 0.05; Figure 6A–D, Supporting Information S2). The top downregulated genes at P12 in P23H retinas (Figure 6A) encode premelanosome protein (*Pmel*) and transmembrane glycoprotein NMB (*Gpnmb*), which are involved in melanin synthesis (Chrystal et al. 2021). After eye opening, inflammation‐associated markers, such as signal transducer and activator of transcription 3 (*Stat3*) and B‐cell lymphoma 3‐encoded protein (*Bcl3*), were prominently upregulated in RP (Figure 6B–D). Inspection of genes involved in the dopaminergic synapse (components taken from the “dopaminergic synapse” KEGG pathway) and other catecholaminergic genes of interest without log2(fold‐change) limits revealed complex system‐wide alterations related to, for instance, catecholaminergic GPCRs, calcium and glutamate signalling and downstream effectors (adjusted *p*‐value < 0.05; Figure 6E–H). RNA‐seq data also further confirmed that both *Maoa* (P30 *p* = 0.007) and *Comt* (P30 *p* = 0.0118, P90 *p* = 0.0034) transcripts are upregulated in adult P23H retinas. The strongest disease‐related upregulation was observed in solute carrier family 6 member 2 (*Slc6a2*, P18 *p* = 0.0491, P30/P90 *p* < 0.0001) followed by protein phosphatase 1 regulatory subunit 1B (*Ppp1r1b*, P30/P90 *p* < 0.0001) and FosB proto‐oncogene (*Fosb*, P18/P30/P90 *p* < 0.0001) gene expression.

## 4 Discussion

The purpose of this study was to characterise alterations in the retinal dopaminergic system during chronic retinal degeneration. Although DA is a key neuromodulator regulating light adaptation, retinal circuitry, and neuronal development and survival, its role in RP progression remains incompletely understood. To address this, we investigated dopaminergic changes in the widely used P23H rhodopsin mutant and rd10 mouse models, representing autosomal dominant and recessive RP, respectively. Our primary focus was on the P23H model as rhodopsin mutations account for a large portion of RP cases, whereas *Pde6b* mutations (modelled for instance with the rd1 and rd10 mice) are rarer (Hartong et al. 2006). Our main finding is an increase in retinal DA in RP retinas of both disease models in both very young and adult mice (Figures 1 and 2). In line with this, previous measurements from retinas of 4‐ to 12‐week‐old rd10 mice housed in standard laboratory‐light conditions have demonstrated elevated DA (Park et al. 2013). In prior research, unaltered retinal DA amounts have been reported in the more severe rd1 (Park et al. 2013) and in a peripherin‐2‐mutated rds mouse model (Nir et al. 2000; Nir and Iuvone 1994). However, RP‐modelling rds mice are albino, which can result in alterations in the dopaminergic system through the lack of tyrosinase enzyme and pigment synthesis, thus complicating comparisons to pigmented animals (Jeffery et al. 1997). In contrast to our results, a study utilising 15‐week‐old RCS rats, which do not have a detectable outer nuclear layer anymore due to advanced degeneration (Ryals et al. 2017), reported lower retinal DA intensity compared with healthy tissue in an immunohistochemistry experiment (Vugler et al. 2007). Notably, our study utilised pigmented mice and focused on earlier stages of RP progression to avoid overt retinal remodelling and reprogramming, including neurotransmitter systems, which is characteristic of RP progression (Pfeiffer et al. 2020). As a limitation, MALDI‐MSI was performed with one sex only due to the low‐throughput nature of the method; however, the UHPLC results indicate that male and female RP mice exhibit similar DA levels (Figure S1).

The increased retinal DA levels observed in P23H and rd10 mice could indicate overactivation of the system and reflect alterations at multiple regulatory levels, including DA synthesis, activity‐dependent vesicular release, reuptake, and metabolic degradation, all of which interact to determine net DA availability and activity. We found increased *Th* gene expression in P23H mouse retinas and a trend towards protein upregulation (Figure 4), which suggests increased capacity for DA synthesis. Furthermore, DA's precursor L‐DOPA tended to be higher in adult RP retinas. However, although the samples were visually assessed for any pigment contamination, these data should be interpreted cautiously. L‐DOPA accumulates strongly in RPE cells, meaning that even minor RPE contamination of samples may affect the measurements.

Importantly, in a snapshot experiment we detected an increase in DA levels in the vitreous humour similar to that in the retinas of P23H mice (Figure 1C). As DA detected in the vitreous likely reflects extracellular overflow following retinal release, this finding suggests increased DA availability outside DACs, consistent with the idea of enhanced dopaminergic activity in RP. Literature shows that while the number of DACs remains normal in rd10 mice, their morphology changes (Ivanova et al. 2016). Furthermore, the function of DACs appears to be altered in retinal degeneration, as their light‐stimulated responses increase with disease progression, although the spontaneous bursting activity decreases in the rd1 mouse (Atkinson et al. 2013). It is important to note that the activity of DACs does not dictate DA release in isolation, and that rod photoreceptor activation may be a major driving force for it (Pérez‐Fernández et al. 2019). Because rod photoreceptor degeneration is the primary hallmark of RP, the consequences of reduced rod‐dependent input to downstream retinal circuitry, particularly to DACs, warrant systematic investigation in future studies. Collectively, these findings raise the possibility that DACs lose autoregulatory control of DA synthesis and release during RP, resulting in elevated retinal DA levels.

Increased dopaminergic activity was further supported by the evident upregulation of enzymes mediating DA metabolism (Figure 5). This was particularly clear with respect to COMT, whose transcript and protein levels, as well as enzyme activity, were significantly elevated (Figures 5B–H and 6E). Furthermore, *Maoa* levels were higher in adult RP retinas as demonstrated by both qPCR (Figure 5A) and RNA sequencing (Figure 6E), and in line with this, a disease‐related trend for increased retinal DOPAC was detected in rd10 samples at P60 (Figure 2B). Overall, it is conceivable that DA's metabolic pathways in the RP retina are upregulated in response to elevated DA content.

The DOPAC/DA ratio is widely used as a proxy for DA turnover, as DOPAC represents the primary MAO‐derived metabolite of DA. A decrease in this ratio was detected in P23H retinas (Figure 1F), particularly in adulthood (P60–90). This ratio change was caused by elevated DA (Figure 1B) as DOPAC was unaltered (Figure 1E), which may point to saturation of the metabolic pathway in P23H mouse retinas. However, while previous studies have mainly assessed retinal DA metabolism using DOPAC levels and the DOPAC/DA ratio (Nir et al. 2000; Park et al. 2013; Pérez‐Fernández et al. 2017, 2019), our comparative analysis of MAO and COMT expression in ocular tissues and the brain suggests that MAO‐mediated metabolism may be secondary in the retina, implying that DOPAC‐based measures alone may not fully capture retinal DA metabolism and that COMT‐dependent pathways may play a larger role (Figure 3). The prominent role of COMT‐mediated metabolism in the retina may relate to developmental similarities between the retina and the frontal cortex, where COMT represents a major pathway for DA clearance (Tunbridge et al. 2007), in contrast to deep brain dopaminergic regions such as the striatum (Evangelou and Alrawashdeh 2016).

To complement our targeted qPCR analyses of dopaminergic enzymes, and to assess broader transcriptional adaptations, we analysed RNA‐seq data from whole‐retina extracts. These data collectively indicate a shift towards enhanced stimulatory catecholaminergic activity in P23H retinas (Figure 6E–G). First, the prominent presence and role of the inhibitory DRD4 in photoreceptors is well established (Deming et al. 2015; Klitten et al. 2008), and the decrease in its gene expression could be a direct consequence of dying rods (Figure 6E), in line with previous research with rd10 mice (Leinonen et al. 2024). More interestingly, a modest increase in *Drd1* expression was noted in P23H retinas, as well as several adrenergic system changes such as the upregulation of alpha‐1D adrenergic receptor (*Adra1d*), a gene encoding an additional stimulatory catecholaminergic receptor, which was upregulated by the disease already at P18. Another upregulated gene suggesting dopaminergic overactivation in P23H retinas is *Ppp1r1b* (starting at P30), encoding DA‐ and cAMP‐regulated neuronal phosphoprotein (DARPP‐32, Figure 6G). DARPP‐32 is present in amacrine cells, horizontal cells and Müller glia cells, and it is activated by phosphorylation via DRD1 as its downstream effector (Svenningsson et al. 2004; Witkovsky et al. 2007; Yan et al. 2006). DARPP‐32 overexpression can compromise RPE function and structure (Cai et al. 2022). Interestingly, the current data additionally demonstrate the downregulation of histidine decarboxylase (*Hdc*) starting at P18 in P23H retinas (Figure 6B–D, Supporting Information S2). This gene produces the rate‐limiting enzyme in the formation of histamine, and genetic knockout of *Hdc* increases dopaminergic signalling, for instance by increasing DA release and DARPP‐32 expression in the brain (Greferath et al. 2014; Koski et al. 2020). Curiously, *Cartpt*, which encodes the CART neuropeptide and is regulated by dopaminergic cAMP signalling pathways that also involve DARPP‐32 in many neuronal contexts (Greengard et al. 1999; Jones and Kuhar 2006), has been shown to be downregulated in rd10 mouse cones after treatment with an inhibitory and retinoprotective catecholaminergic drug cocktail (Leinonen et al. 2024). Finally, the most striking upregulation in P23H retinas was seen in *Slc6a2* (Figure 6E), which encodes norepinephrine transporter (NET). Retinal expression of the gene encoding DA transporter (DAT, *Slc6a3*) seems negligible (Supporting Information S3), which suggests that retinas could utilise NET for DA and catecholamine reuptake similarly to brain regions that may lack DAT (Morón et al. 2002). However, it should be noted that *Slc6a2* transcript levels appear very low in the WT retina in our data (Supporting Information S3), and thus, it may not have a major role in catecholamine uptake in normal retinal physiology.

An important question is whether the apparent increase in dopaminergic, and more broadly catecholaminergic activity in RP influences disease progression. Notably, retinal DA levels were already elevated before eye opening (P12) in the P23H mouse model (Figure 1B). Although absolute DA levels were low at this point in both WT and P23H mice, this observation suggests that the increase is not solely a consequence of degeneration‐associated retinal remodelling. Anatomical retinal degeneration has not yet started at this point, as initial degenerative rod outer segment changes appear at P15 when the first markers of photoreceptor apoptosis also occur (Chiang et al. 2015). Together with the increased DA concentrations, the trend towards elevated DOPAC (Figure 1E), and on the other hand decreased L‐DOPA at P12 (Figure 4A), could point towards increased DA synthesis and turnover already before any rod loss in P23H retinas. Considering that DA and melanin synthesis share a common source within tyrosine utilisation (Jeffery et al. 1997), the marked reduction in *Gpnmb* and *Pmel* expression in P23H retinas at P12 (Figure 6A) may reflect altered pigment‐related developmental pathways associated with early dopaminergic changes that could influence disease onset. As discussed above, additional alterations in the catecholaminergic system emerge after eye opening as retinal degeneration progresses (Figures 1 and 4, 5, 6). Evidence from multiple studies shows that inhibition of the catecholaminergic system via DRD2 or adrenergic ADRA2 agonism, or alternatively ADRA1 or beta‐receptor antagonism, decelerates retinal degeneration in vivo (Chen et al. 2013, 2016; Kanan et al. 2019; Leinonen et al. 2019, 2024; Orban et al. 2018; Shibagaki et al. 2015; Tanaka et al. 2021), which could indicate that elevated DA is connected to harmful system overactivation in disease. Moreover, besides the potential direct detrimental effects of excess DA (Cai et al. 2022; Meiser et al. 2013), increased DA could impact RP progression also via effects on cyclic guanosine monophosphate (cGMP), as inhibition of DA signalling with 6‐OHDA‐mediated DAC depletion has been shown to decrease the elevated retinal cGMP levels in RP (Zhang et al. 2014). Normal cGMP levels are crucial for normal photoreceptor physiology, but pathologically increased levels exacerbate RP (Paquet‐Durand et al. 2009; Power et al. 2020; Sothilingam et al. 2015; Vighi et al. 2018).

The present findings, together with previous pharmacological evidence, support an association between increased catecholaminergic activity and RP progression, but whether this relationship is causal remains unresolved. More targeted studies could address this through receptor‐specific pharmacological blockade or inducible/cell‐type‐specific deletion of dopaminergic or adrenergic receptors. Constitutive deletion of several DA receptors did not protect photoreceptors in rd1 mice (Ogilvie et al. 2009), highlighting potential effects of disease model, developmental compensation, and intervention timing. Complementary approaches could test whether increasing dopaminergic activity, for example with L‐DOPA or manipulation of DA metabolism, accelerates degeneration, or whether intravitreal 6‐OHDA‐mediated depletion of DACs slows disease progression, while acknowledging the non‐specific effects of this neurotoxic approach. Such interventions should be examined before and after disease onset to distinguish effects on disease initiation from progression.

In conclusion, this work demonstrates elevated retinal DA levels and metabolism, together with broader alterations in the catecholaminergic system, indicating increased dopaminergic activity in RP retinas during postnatal eye development and adulthood. Further mechanistic studies are warranted to determine the functional consequences of this apparent dopaminergic overactivation.

## Author Contributions

**Katri Vainionpää:** conceptualization, project administration, investigation, methodology, validation, data curation, formal analysis, visualization, writing – original draft. **Eerik Lappalainen:** investigation. **Anna Nilsson:** investigation, methodology, validation, visualization, writing – review and editing. **Ida Pavela:** investigation. **Tommi Torsti:** methodology. **Umair Seemab:** software. **Elina Mäkinen:** investigation. **Reza Shariatgorji:** methodology, investigation, validation. **Per E. Andrén:** resources. **Aaro Jalkanen:** investigation, writing – review and editing. **Markus M. Forsberg:** resources, supervision, writing – review and editing. **Henri Leinonen:** investigation, validation, formal analysis, visualization, resources, funding acquisition, supervision, writing – review and editing.

## Funding

This study was funded by the Research Council of Finland (grant 346295), Jane & Aatos Erkko Foundation, and the Federation of European Biochemical Societies. H.L. was additionally supported by grants from the Emil Aaltonen Foundation (Emil Aaltosen Säätiö), Sigrid Jusélius Foundation (Sigrid Juséliuksen Säätiö), Finnish Eye and Tissue Bank Foundation (Silmä‐ ja kudospankkisäätiö), Retina Registered Association Finland (Retina ry), and Sokeain Ystävät/De Blindas Vänner Registered Association. K.V. was funded by the UEF Doctoral School and Orion Research Foundation sr.

## Conflicts of Interest

The authors declare no conflicts of interest.

## Supporting information


**Data S1:** jnc70552‐sup‐0001‐DataS1.xlsx.

**Data S2:** jnc70552‐sup‐0002‐DataS2.xlsx.

**Data S3:** jnc70552‐sup‐0003‐DataS3.xlsx.

**Figure S1:** jnc70552‐sup‐0004‐FigureS1.docx.

## Data Availability

Data is available as Supporting Information. RNA‐seq dataset is publicly available (GEO accession number GSE334684). All code and analysis scripts are publicly available in the GitHub repository (https://github.com/UmairSeemab/RNAseqData).
