# Supplementary material for: Altered Retinal Dopamine Homeostasis in Murine Models of Retinitis Pigmentosa
Source: J Neurochem. 2026 Sep 18;170(9):e70552. doi: 10.1111/jnc.70552 (PMC13587835; doi:10.1111/jnc.70552)
Supplement: Supplementary file 1 [file JNC-170-e70552-s003.docx]

Supplementary information for

**Altered retinal dopamine homeostasis in murine models of retinitis pigmentosa**

Katri Vainionpää^1^, Eerik Lappalainen^1^, Anna Nilsson^2^, Ida Pavela^1^, Tommi Torsti^1^, Umair Seemab^1^, Elina Mäkinen^1^, Reza Shariatgorji^2^, Per Andrén^2^, Aaro Jalkanen^1^, Markus Forsberg^1^, Henri Leinonen^1*^

^1^ School of Pharmacy, Faculty of Health Sciences, University of Eastern Finland, Kuopio 70211, Finland.

^2^ Department of Pharmaceutical Biosciences, Spatial Mass Spectrometry, Science for Life Laboratory, Uppsala University, SE-75124 Uppsala, Sweden.

*Correspondence: Henri Leinonen, University of Eastern Finland, School of Pharmacy, Yliopistonrinne 3, Kuopio 70210, Finland, tel. +358 50 430 3196, email: [henri.leinonen@uef.fi](mailto:henri.leinonen@uef.fi)

**This file includes:**

Supplementary figures 1-5

Legends for Supplementary data files 1-3.

 **Supplementary figure 1. Sex-segregation of retinal dopamine (DA), 3,4-dihydroxyphenylacetic acid (DOPAC), and L-3,4-dihydroxyphenylalanine (L-DOPA) content and catechol-O-methyltransferase (COMT) enzyme activity data from P23H, rd10 and wild-type (WT) mice.** This figure relates to main figures 1,2, 4 and 5. A-F) Retinal DA, (panels A and B), DOPAC (panels C and D) and L-DOPA (E and F) concentrations in retinas collected in light-adapted (LA, adaptation state in all P23H experiments) or dark-adapted (DA) state at different timepoints. Analyte concentrations (ng/g tissue) are presented as mean ± SD. G) COMT activity results (pmol/min/mg protein) in all WT (P60-90), and LA P23H (P90), LA rd10 and DA rd10 (P60) retinas, mean ± SD. Data normality and statistical tests are presented in Supplementary data file 1.

**Supplementary figure 2. Full western blot images used in the study from experiments with healthy wild-type brain cortex (CTX) and striatum (STR), retina (RET) and eye cup (EC) samples.** This figure relates to main figure 3. Blots present A) tyrosine hydroxylase (TH), B) catechol-o-methyltransferase (COMT, membrane-bound (MB) and soluble (S) form), or C) monoamine oxidase A (MAO-A; one EC lane excluded as an outlier due to unsure chemiluminescence inducement) staining with healthy wild-type (WT) samples. Results were normalised with Ponceau S total protein staining (D).

**Supplementary figure 3. Full western blot images used in the study from experiments with retinitis pigmentosa retinas.** This figure relates to main figures 4 and 5. Blots present A-B) tyrosine hydroxylase (TH) or C-D) catechol-o-methyltransferase (COMT, membrane-bound (MB) and soluble (S) form) staining with P23H (panels A, C), rd10 (B, D) and wild-type (WT) samples. Results were normalised with Ponceau S total protein staining (E-F).

**Supplementary figure 4. Representative immunohistochemistry images of wild-type (WT) and P23H mouse retinal cryosections.** Each row represents three biological replicates. A) Samples stained with tyrosine hydroxylase (TH, red) antibody and nuclei marker 4′,6-diamidino-2-phenylindole (DAPI, blue). B) Samples stained with monoamine oxidase A (MAO-A, red), catechol-O-methyltransferase (COMT, green) antibodies, and DAPI. This supplementary figure relates to main figures 4 and 5.

**Supplementary figure 5. Quality control and utilization of retinal layer separation (ReLayS) technique to confirm inner retinal localization of genes encoding dopaminergic enzymes in healthy wild-type (WT) retinas.**  Separation of the photoreceptor ciliary segments (PR), outer nuclear layer (ONL) and inner retina (IR) was evaluated by measuring A) phosphodiesterase 6A (*Pde6a*; rod marker), B) Purkinje Cell Protein 2 (*Pcp2;* bipolar cells marker), and C) glial fibrillary acidic protein (*Gfap*: mainly astrocyte marker in WT retina) mRNA expression with quantitative polymerase chain reaction (qPCR). After, dopaminergic target genes D) tyrosine hydroxylase (*Th*), E) monoamine oxidase A (*Maoa*), and F) catechol-O-methyltransferase (*Comt*) were assessed. Data is presented as log10(2^-ΔΔCt^), mean ± SD, and the asterisks indicate significant one-way ANOVA (panels A-E) or Kruskal-Wallis test (panel F) results, * = p < 0.05, ** = p < 0.01, *** = p < 0.001 and **** = p < 0.0001. Data normality and statistical tests are presented in Supplementary data file 1. G) Representative images from a hematoxylin-eosin staining of ReLayS samples. Left panel: After ReLays method. Right panel: Intact full retina. This supplementary figure relates to main figures 4 and 5.

**Supplementary data 1** (separate file). Source data file.

**Supplementary data 2** (separate file). Bulk RNA-seq statistics.

**Supplementary data 3** (separate file). Raw readcounts from bulk RNA-seq.
